# Supplementary material for: Proteome dynamics during homeostatic scaling in cultured neurons
Source: eLife. 2020 Apr 2;9:e52939. doi: 10.7554/eLife.52939 (PMC7117909; doi:10.7554/eLife.52939)
Supplement: Transparent reporting form [file elife-52939-transrepform.docx]

***eLife’s* transparent reporting form**

We encourage authors to provide detailed information *within their submission* to facilitate the interpretation and replication of experiments. Authors can upload supporting documentation to indicate the use of appropriate reporting guidelines for health-related research (see [EQUATOR Network](http://www.equator-network.org/%20)), life science research (see the [BioSharing Information Resource](https://biosharing.org/)), or the [ARRIVE guidelines](http://www.plosbiology.org/article/info:doi/10.1371/journal.pbio.1000412) for reporting work involving animal research. Where applicable, authors should refer to any relevant reporting standards documents in this form.

If you have any questions, please consult our Journal Policies and/or contact us: [editorial@elifesciences.org](mailto:editorial@elifesciences.org).

**Sample-size estimation**

- You should state whether an appropriate sample size was computed when the study was being designed
- You should state the statistical method of sample size computation and any required assumptions
- If no explicit power analysis was used, you should describe how you decided what sample (replicate) size (number) to use

Please outline where this information can be found within the submission (e.g., sections or figure legends), or explain why this information doesn’t apply to your submission:

The number of biological (and technical) replicates are provided in the corresponding figure legends and/or Materials and Methods section. We have performed no calculations for which a sample-size estimation is required.

**Replicates**

- You should report how often each experiment was performed
- You should include a definition of biological versus technical replication
- The data obtained should be provided and sufficient information should be provided to indicate the number of independent biological and/or technical replicates
- If you encountered any outliers, you should describe how these were handled
- Criteria for exclusion/inclusion of data should be clearly stated
- High-throughput sequence data should be uploaded before submission, with a private link for reviewers provided (these are available from both GEO and ArrayExpress)

Please outline where this information can be found within the submission (e.g., sections or figure legends), or explain why this information doesn’t apply to your submission:

Dynamic SILAC experiments: see Methods section

Electrophysiological recordings: see legend of Figure 1 – Figure Supplement 2

WB analysis of selected candidates: see legend of Figure 1 – Figure Supplement 1

AHA labeling and WB analysis: see legend of Figure 3A

eIF2α phosphorylation: see legend of Figure 3B

Proteasome activity assay: see legend of Figure 3 – Figure Supplement 3A

**Statistical reporting**

- Statistical analysis methods should be described and justified
- Raw data should be presented in figures whenever informative to do so (typically when N per group is less than 10)
- For each experiment, you should identify the statistical tests used, exact values of N, definitions of center, methods of multiple test correction, and dispersion and precision measures (e.g., mean, median, SD, SEM, confidence intervals; and, for the major substantive results, a measure of effect size (e.g., Pearson's r, Cohen's d)
- Report exact p-values wherever possible alongside the summary statistics and 95% confidence intervals. These should be reported for all key questions and not only when the p-value is less than 0.05.

Please outline where this information can be found within the submission (e.g., sections or figure legends), or explain why this information doesn’t apply to your submission:

Processing and analysis of dynamic SILAC data: see Methods section.

Processing and analysis of data obtained from electrophysiological recordings: see Methods section and legend of Figure 1 – Figure Supplement 2.

AHA labeling and WB analysis of nascent proteins: see legend of Figure 3A.

eIF2α phosphorylation: see legend of Figure 3B.

uORF analysis: see Methods section and legend of Figure 3C.

Over-representation analysis: see legends of Figure 6, Figure 6 – Figure Supplement 1.

GO-over-representation analysis: see Methods section.

Proteasome activity assay: see legend of Figure 3 – Figure Supplement Figure 3A

Temporal clustering: see Methods section

(For large datasets, or papers with a very large number of statistical tests, you may upload a single table file with tests, Ns, etc., with reference to sections in the manuscript.)

**Group allocation**

- Indicate how samples were allocated into experimental groups (in the case of clinical studies, please specify allocation to treatment method); if randomization was used, please also state if restricted randomization was applied
- Indicate if masking was used during group allocation, data collection and/or data analysis

Please outline where this information can be found within the submission (e.g., sections or figure legends), or explain why this information doesn’t apply to your submission:

n/a

**Additional data files (“source data”)**

- We encourage you to upload relevant additional data files, such as numerical data that are represented as a graph in a figure, or as a summary table
- Where provided, these should be in the most useful format, and they can be uploaded as “Source data” files linked to a main figure or table
- Include model definition files including the full list of parameters used
- Include code used for data analysis (e.g., R, MatLab)
- Avoid stating that data files are “available upon request”

Please indicate the figures or tables for which source data files have been provided:

All proteomics raw data and database search results associated with the manuscript have been uploaded to the PRIDE online repository with accession number PXD016004.

Figure 2 – Source Data 1: Protein regulation during homeostatic up- and down-scaling.

Figure 4 – Source Data 1: Synaptic protein regulation during homeostatic down-scaling.

Figure 5 – Source Data 1: Synaptic protein regulation during homeostatic up-scaling.

Figure 6 – Source Data 1: Changes in protein abundance during homeostatic up- and down-scaling.

Figure 8 – Source Data 1: Temporal protein regulation during homeostatic up- and down-scaling.

Supplementary Data 1: GO term over-representation of proteins with distinct regulation.

Supplementary Data 2: MS settings.

Supplementary Data 3: Sample overview and MaxQuant search parameters.
